# An Interaction between a *FNDC5* Variant and Obesity Modulates Glucose Metabolism in a Chinese Han Population

**DOI:** 10.1371/journal.pone.0109957

**Published:** 2014-11-04

**Authors:** Shanshan Tang, Rong Zhang, Feng Jiang, Jie Wang, Miao Chen, Danfeng Peng, Jing Yan, Yuqian Bao, Cheng Hu, Weiping Jia

**Affiliations:** Shanghai Diabetes Institute, Department of Endocrinology and Metabolism, Shanghai Key Laboratory of Diabetes Mellitus, Shanghai Clinical Center for Diabetes, Shanghai Jiao Tong University Affiliated Sixth People’s Hospital, Shanghai, PR China; Northeast Ohio Medical University, United States of America

## Abstract

**Background:**

To investigate the impact of common variants of *FNDC5* on type 2 diabetes and clinical traits related to glucose metabolism in a large Chinese population sample.

**Methods:**

Three tagging single nucleotide polymorphisms within the region of the *FNDC5* gene were selected and genotyped in 6822 participants. Detailed clinical investigations and biochemistry measurements were carried out in all of the participants. Subjects without diabetes were classified into normal weight and overweight/obese subgroups according to body mass index (BMI).

**Results:**

None of the SNPs were associated with either the risk of type 2 diabetes in all of the participants or with any of the clinical quantitative traits in the controls with normal glucose regulation. Subgroup analysis showed that in controls with normal weight (BMI <25 kg/m^2^), the rs16835198 major allele G was significantly associated with fasting insulin levels, and that each additional copy of the allele resulted in a 0.0178 mU/L increment of the values (*p* = 0.046). Moreover, after adjusting for confounding variables, there were trends towards correlation of rs16835198 with HOMA-insulin resistance (HOMA-IR) (*p = *0.057) and low-density lipoprotein cholesterol (LDL-C) levels (*p = *0.083). In overweight/obese subjects (BMI ≥25 Kg/m^2^), we noted rs16835198 showed trends towards association with fasting insulin (*p = *0.057) and HOMA-IR levels (*p = *0.091), both of which declined with additional copies of the major allele G. Moreover, rs16835198 was significantly associated with high-density lipoprotein cholesterol (HDL-C) levels (*p = *0.013), and HOMA-β cell function (*p = *0.028) in the overweight/obese subjects. Finally, we observed a significant interaction between BMI-rs16835198 and fasting insulin levels in the control group (*p = *0.003).

**Conclusions:**

Our data indicate that the effect of the common *FNDC5* SNP rs16835198 on fasting insulin was significantly modified by BMI in the Chinese Han population.

## Introduction

Diabetes is a major public health concern, and changes in diet and lifestyle have accelerated the rise in the incidence of this disease to pandemic proportions in many countries worldwide, including China. According to the national epidemiological survey conducted in 2008, China has the largest number of diabetics in any single country [1], of which 90% have type 2 diabetes. It is acknowledged that insulin resistance plays an important role in the pathogenesis of diabetes as well as obesity. In recent decades, with the global prevalence of obesity rising, the burden of chronic metabolic disorders has been boosted even further [2].

Physical exercise is recognized not only as an indispensable component of lifestyle intervention but also, increasingly, as a cornerstone for non-pharmacological therapy in ameliorating obesity and insulin resistance [3], [4]. As the largest organ in the body, skeletal muscle is increasingly considered an important endocrine organ that interacts with adipose tissue, pancreas, and liver to modulate metabolism through the beneficial effects of exercise [5]–[7]. In addition, skeletal muscle secretes metabolically active myokines such as irisin [8], which is encoded by the *FNDC5* (*FRCP2*, *PeP*) gene in both mice and humans [9], [10]. Irisin undergoes proteolytic cleavage to yield a mature 112 aa polypeptide, which is secreted in response to physical exercise [8]. Irisin is expressed in fat, kidney, lung, and liver [11], with peak expression levels in skeletal muscle, where it is regulated by peroxisome proliferator-activated receptor γ (PPARγ) coactivator 1α (PGC1-α), an exercise-responsive mediator that is involved in energy metabolism [8]. Irisin has been shown in mice to induce the transformation of white adipose tissue to brown adipose tissue by induction of the *Ucp1* and *Cidea* genes, implying its potential as therapeutic for combat obesity, diabetes and other disease could be ameliorate by exercise [8], [12], [13]. Up to now, two previous genetic studies have investigated the association of common SNPs in *FNDC5* with insulin sensitivity and glucose metabolism [14], [15]. In addition, although irisin is a myokine known to be closely related with obesity, its impact on the relationship between glycolipid metabolism and BMI is not clearly defined. Given the lack of data implicating *FNDC5* variants in human metabolic disease, we set out to examine the association between *FNDC5* variants and a range of metabolic parameters in a large Chinese Han population.

## Materials and Methods

### Ethics statement

The study was approved by the Ethics Committee of Shanghai Jiao Tong University Affiliated Sixth People’s Hospital and in accordance with the Declaration of Helsinki. Written informed consent was obtained from each participant.

### Subjects recruitments

The overall study group totally comprised 6822 participants of Han Chinese ancestry residing in Shanghai. Detailed information regarding this study population was described in detail previously [16]–[18]. Among them, 3410 participants were unrelated individuals with type 2 diabetes, defined according to 1999 WHO criteria (fasting plasma glucose ≥7.0 mmol/l and/or 2-h postchallenge plasma glucose ≥11.1 mmol/l), and who were recruited from the clinical inpatient database of Shanghai Diabetes Institute [19]. The remaining 3412 participants were unrelated controls with normal glucose tolerance as assessed by 75 g oral glucose tolerant tests (OGTTs), and a negative family history of diabetes, and who were selected from community-based epidemiological studies of diabetes and related metabolic diseases. Using 1999 WHO criteria [20], BMI was used to divide individuals with normal blood glucose into two groups, namely: normal weight (BMI<25 Kg/m^2^), and overweight/obese (BMI≥25 Kg/m^2^). The clinical characteristics of all participants are shown in Table 1.

**Table 1:** Clinical characteristics of the study sample population.

	Cases of type2 diabetes	Controls	Controls	
			Normal weight	Overweight/obesity
Samples(n)	3410	3412	2387	1022
Male/female (n)	1812/1597	1364/2048	920/1467	442/580
Age (years)	60.33±12.49	51.41±14.39	50.53±14.86	53.45±12.96
BMI (kg/m^2^)	24.20 (22.00,26.60)	23.23 (21.27,27.68)	22.07 (20.55,23.45)	26.78 (25.81,28.23)
Fasting plasma glucose(mmol/L)	12.78 (9.00,16.00)	5.02 (4.70,5.40)	5.00 (4.64,5.38)	5.10 (4.73,5.46)
2-h plasma glucose (mmol/L)	17.00 (13.00,22.00)	5.42 (4.60,6.30)	5.30 (4.53,6.20)	5.70 (4.80,6.60)
Total cholesterol (mmol/L)	4.70 (4.00,5.50)	4.70 (4.04,5.35)	4.60 (3.97,5.30)	4.86 (4.20,5.50)
Triglyceride(mmol/L)	1.49 (0.99,2.18)	1.25 (0.87,1.82)	1.13 (0.80,1.64)	1.61 (1.11,2.26)
HDL-C (mmol/L)	1.11 (0.94,1.33)	1.33 (1.13,1.51)	1.36 (1.18,1.55)	1.25 (1.05,1.43)
LDL -C (mmol/L)	2.97 (2.42,3.57)	3.04 (2.49,3.61)	2.98 (2.42,3.53)	3.20 (2.64,3.80)

Data are shown as n or median (interquartile range).

BMI, body mass index; HDL-C, high-density lipoprotein cholesterol; LDL-C, low-density lipoprotein cholesterol.

### Clinical measurements

All participants underwent a detailed clinical investigation as described previously [21], [22]. In summary, anthropometric parameters such as height, weight, waist and hip circumference were measured. Body mass index (BMI) was calculated as weight in kilograms divided by height in meters squared. Controls were subjected to standard OGTTs involving administration of 75 g glucose in the morning after an overnight fast. Blood samples were obtained at baseline and 2 h time points during the OGTT. Plasma glucose, serum insulin and serum lipid profiles were also measured. Insulin resistance index and β cell function were estimated using the homeostasis model assessment (HOMA), which was calculated using fasting plasma glucose and insulin levels [23]. In addition, Insulin sensitivity from the OGTT was also estimated according to the insulin sensitivity index (ISI) proposed by Gutt *et al.* [24]: ISI = [75000+ (fasting plasma glucose −2 h plasma glucose) × 0.19×weight]/120/mean plasma glucose/log_10_ mean insulin.

### Single nucleotide polymorphism (SNP) selection and genotyping

Using the HapMap Phase III (release 27) Han Chinese database and a threshold of r^2^≥0.8, we selected three tagging SNPs in a region encompassing the *FNDC5* gene and its 10 kb up- and downstream sequences. These three tagging SNPs tagged 100% of SNPs (5/5 SNPs in the HapMap Chinese Han sample) with a minor allele frequency (MAF) of >0.05. All SNPs were genotyped by primer extension of multiplex products with detection by matrix-assisted laser desorption ionization–time of flight mass spectroscopy, using a MassARRAY Compact Analyzer (Sequenom, San Diego, CA, USA) with an overall call rate of 99.6%.

### Statistical analysis

For each variant, the Hardy-Weinberg equilibrium test was performed separately in the cases and controls prior to association analysis. SNPs failing this test (*p*<0.05 in the controls) were excluded. Pairwise linkage disequilibrium including |D’| and r^2^ was estimated using Haploview (version 4.2). Allele and genotype distributions between the cases and control subjects were compared with χ^2^ test or logistic regression [25], and odds ratios (ORs) with 95% confidence intervals (CIs) were calculated. All skewly distributed quantitative traits [including fasting plasma glucose, 2 h plasma glucose, fasting insulin, 2 h insulin, triglycerides, total cholesterol, low-density lipoprotein cholesterol (LDL-C), high-density lipoprotein cholesterol (HDL-C), estimated ISI, HOMA for β-cell function and insulin resistance] were logarithmically transformed to approximate univariate normality. Quantitative traits were analyzed in the control group by linear regression under an additive genetic model adjusted for age, gender and BMI as potential confounders. Both BMI-pooled analysis and BMI-specific analysis were conducted. All statistical analyses were performed using SAS for Windows (version 8.0; SAS Institute, Cary, NC, USA). The interaction analysis was accounted using PLINK (version 1.05). A two tailed p-value of 0.05 was considered statistically significant.

Quanto was used to calculate the statistical power [26]. Based on the assumption that the population risk was 9.6%, and a two-side α of 0.05, for SNPs in our sample with a minor allele frequency over 0.2, we had over 80% power to detect the minimum OR of 1.13 under the additive model.

## Results

**Figure 1:**
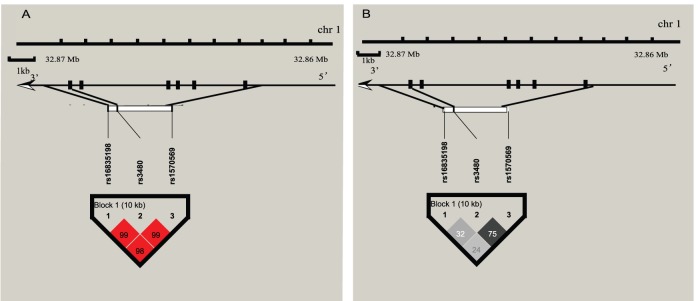
Linkage disequilibrium maps for SNPs genotyped in the region of the *FNDC5* gene. A. Shades of red demonstrate the strength of the pairwise linkage disequilibrium based on D’ and numbers represent the value of D’ expressed as a percentage. B. Shades of grey show the strength of the pairwise linkage disequilibrium based on r^2^ and numbers indicate the value of r^2^ expressed as a percentage.

All the three SNPs were in Hardy-Weinberg equilibrium in the control population (*p*>0.05). Linkage disequilibrium analysis for these SNPs was shown in Figure 1. One haplotype block was constructed in this region. Single SNP association analysis failed to detect a significant association between any of the three SNP and type 2 diabetes, with a minimum *p* value of 0.265 for rs3480. Similarly, logistic regression analysis found no association between any SNP and type 2 diabetes (Table 2). To determine the effect of these SNPs on clinical characteristics in the control participants, we next performed genotype-phenotype analyses using an additive genetic model. As shown in the Supporting Information (Table S1 in File S1), none of the SNPs were significantly associated with quantitative traits related to glucose and lipid metabolism in the control population.

**Table 2:** Associations of *FNDC5* SNPs with type 2 diabetes.

SNP	Chr. Position(Build 104)	Major/minorallele	Cases of type2 diabetes (n = 3410)		Controls (n = 3412)		OR for major allele(95%CI)	*p* value for majorallele	OR for genotype(95%CI)	*p* value forgenotype (*p* *)
			Major allelefrequencies	Genotypecount 11/12/22#	Major allelefrequencies	Genotypecount 11/12/22#				
rs16835198	33326681	G/T	0.518	929/1661/807	0.519	899/1735/768	0.995(0.930,1.064)	0.880	0.995(0.930,1.064)	0.880(0.929)
rs3480	33328165	A/G	0.734	1855/1275/267	0.742	1874/1306/225	0.957(0.887,1.034)	0.265	0.958(0.888,1.034)	0.269(0.205)
rs1570569	33336956	G/T	0.782	2088/1126/175	0.790	2121/1122/153	0.956(0.930,1.064)	0.286	0.957(0.882,1.038)	0.290(0.240)

The additive model was used in the association analyses between genotype and type 2 diabetes.

# 11, major allele homozygotes; 12, heterozygotes; 22, minor allele homozygotes.

*Adjusted for age, gender and BMI.

**Figure 2:**
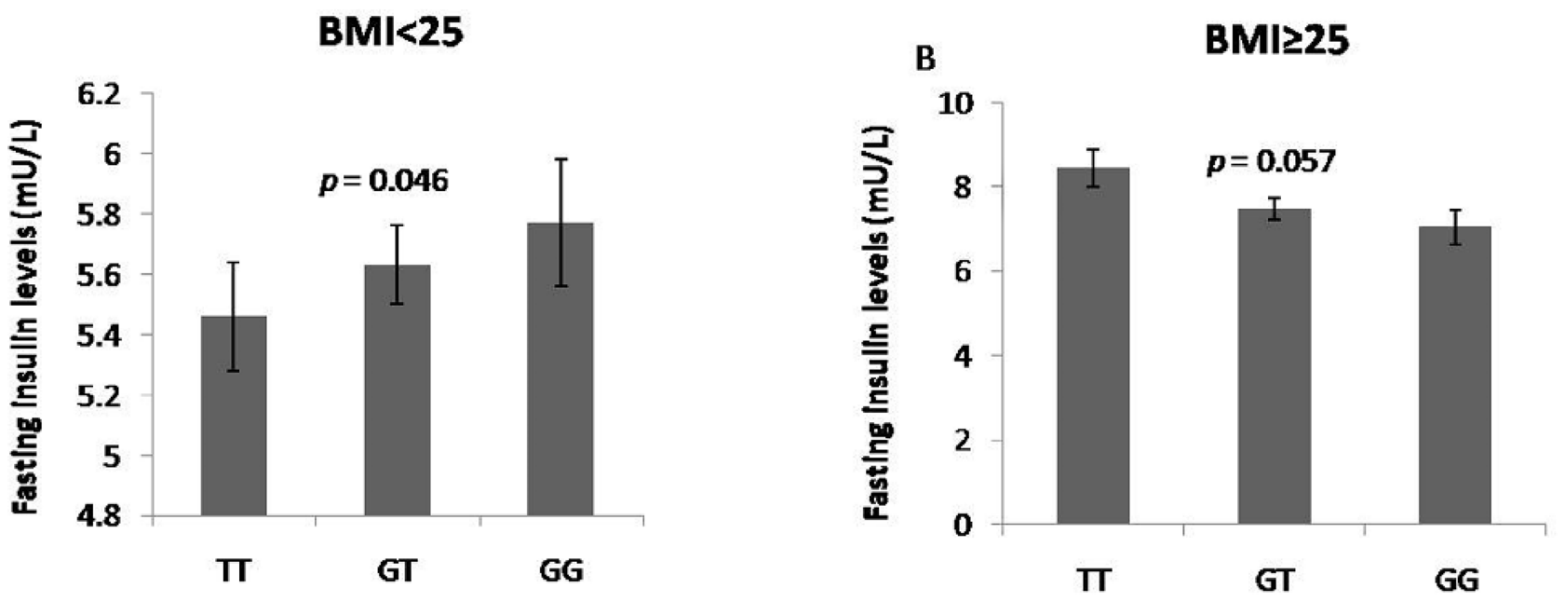
Association between the SNP rs16835198 and fasting insulin levels in BMI-specific controls, under the additive genetic model. The *p* value for interaction between rs16835198-BMI on fasting insulin was 0.003. A. Normal weight controls (BMI <25). B. Overweight/obesity controls (BMI ≥25).

To examine the effects of *FNDC5* SNPs on glycolipid metabolism parameters in subjects with different BMI, we then analyzed the distribution of these SNPs in two BMI-based subgroups of individuals with normal glucose regulation, namely, overweight/obese or normal weight. In the normal weight subgroup (BMI <25 Kg/m^2^), rs16835198 major allele G was significantly associated with a 0.0178 mU/L increment in fasting insulin levels for each additional allele copy (*p = *0.046). Moreover, after adjustment for confounding variables, this SNP exhibited trends towards association with HOMA-IR (*p = *0.057) and serum LDL-C levels (*p = *0.083) in the same subgroup (Table 3). However, opposite association was observed in overweight/obese subgroup (BMI ≥25 Kg/m^2^) subgroup, with each additional copy of major allele (G) exhibiting lower values than TT homozygotes (*p = *0.057 for fasting insulin, *p = *0.091 for HOMA-IR, respectively). Moreover, rs16835198 was also significantly associated with HDL-C and HOMA-β cell function levels in the overweight/obese subgroup both before (*p = *0.013, 0.028, respectively) and after (*p = *0.001, 0.025, respectively) adjusting for these covariates (Table 4). Finally, we noted a conflict between the SNP rs16835198 and fasting insulin levels in the different subgroups (Figure 2). When we tested the SNP-BMI interaction in participants with normal glucose regulation, a significant interaction between rs16835198-BMI and fasting insulin levels was observed (*p = *0.003). Results regarding the association between the other two SNPs and clinical traits in BMI-specific subgroups are shown in Supporting Information (Table S2 and S3 in File S1).

**Table 3:** Association analyses of rs16835198 with clinical characteristics in normal weight controls.

rs16835198	TT (n = 537)	GT (n = 1192)	GG(n = 650)	β	SE	*p*	*p* *
Age (years)	50.36±15.25	50.51±14.42	50.66±15.39	0.1513	0.4327	0.727	/
BMI (kg/m^2^)	22.06 (20.52,23.34)	22.10 (20.58,23.45)	22.07(20.50,23.52)	0.0014	0.0012	0.261	/
Fasting plasma glucose(mmol/L)	5.03 (4.70,5.40)	5.00 (4.62,5.35)	5.00 (4.65,5.35)	-0.0004	0.0013	0.739	0.572
2-h plasma glucose (mmol/L)	5.49(4.54,6.21)	5.30 (4.52,6.20)	5.20 (4.55,6.12)	−0.0027	0.0028	0.328	0.211
Fasting insulin (mU/L)	5.46(3.94,7.65)	5.63 (4.07,7.73)	5.77 (3.98,7.94)	0.0178	0.0089	**0.046**	*0.059*
2-h insulin (mU/L)	24.21(15.45,43.63)	25.79 (13.50,41.75)	23.05 (14.08,41.29)	−0.0049	0.0124	0.691	0.498
Total cholesterol (mmol/L)	4.60(3.97,5.30)	4.65 (4.00,5.30)	4.55 (3.91,5.26)	−0.0027	0.0029	0.339	0.190
Triglyceride (mmol/L)	1.12 (0.83,1.64)	1.13 (0.81,1.62)	1.13(0.77,1.66)	−0.0027	0.0066	0.684	0.514
HDL-C (mmol/L)	1.37 (1.21,1.55)	1.36 (1.19,1.55)	1.35(1.15,1.56)	−0.0033	0.0028	0.238	0.198
LDL-C (mmol/L)	2.98(2.45,3.48)	3.01 (2.46,3.58)	2.91 (2.36,3.53)	−0.0060	0.0039	0.119	*0.057*
HOMA-IR	1.17 (0.85,1.71)	1.23 (0.86,1.66)	1.23 (0.83,1.72)	0.0172	0.0093	*0.064*	*0.083*
HOMA-B	80.00 (54.90,121.33)	84.81 (60.67,128.29)	87.07 (61.36,126.40)	0.0158	0.0102	0.124	0.128
Gutt-ISI	691.58(563.64,889.09)	697.61(573.72,903.39)	707.89(583.82,886.08)	0.0013	0.0054	0.813	0.589

Data are shown as mean±SD or median (interquartile range).

BMI, body mass index; HDL-C, high-density lipoprotein cholesterol; LDL-C, low-density lipoprotein cholesterol; HOMA-IR, homeostasis assessment model of insulin resistance;

HOMA-B, homeostasis assessment model of β-cell function; Gutt-ISI, insulin sensitivity index proposed by Gutt.

*p* values <0.05 are shown in bold, *p* values <0.1 are shown in italics.

*Adjusted for age, gender and BMI.

**Table 4:** Association analyses of rs16835198 with clinical characteristics in overweight and obese controls.

rs16835198	TT (n = 229)	GT (n = 543)	GG(n = 248)	β	SE	*p*	*p* *
Age (years)	53.69±13.01	53.66±12.78	52.68±13.34	−0.5141	0.5938	0.387	/
BMI (kg/m^2^)	26.77 (25.73,28.38)	26.76 (25.78,28.13)	26.81(25.90,28.26)	−0.0563	0.0954	0.555	/
Fasting plasma glucose(mmol/L)	5.06 (4.70,5.40)	5.10 (4.77,5.44)	5.10 (4.73,5.48)	0.0007	0.0020	0.706	0.613
2-h plasma glucose (mmol/L)	5.70(4.90,6.57)	5.80 (4.80,6.60)	5.66 (4.75,6.70)	0.0025	0.0042	0.549	0.334
Fasting insulin (mU/L)	8.43(5.83,11.38)	7.47 (5.22,10.50)	7.03 (5.04,10.18)	−0.0267	0.0140	*0.057*	*0.054*
2-h insulin (mU/L)	31.34(18.99,60.36)	37.22 (19.77,56.06)	35.34 (19.52,60.18)	0.0125	0.0197	0.528	0.437
Total cholesterol (mmol/L)	4.90(4.20,5.52)	4.85 (4.18,5.54)	4.81 (4.30,5.40)	0.0018	0.0042	0.669	0.469
Triglyceride (mmol/L)	1.64 (1.09,2.26)	1.58 (1.10,2.25)	1.64(1.16,2.30)	−0.0029	0.0108	0.793	0.580
HDL-C (mmol/L)	1.22 (1.00,1.44)	1.24 (1.06,1.41)	1.29(1.10,1.46)	0.0113	0.0045	**0.013**	**0.001**
LDL-C (mmol/L)	3.24(2.62,3.81)	3.18 (2.63,3.80)	3.20 (2.71,3.79)	0.0022	0.0059	0.707	0.550
HOMA-IR	1.89 (1.26,2.46)	1.67 (1.12,2.40)	1.59 (1.12,2.24)	−0.0245	0.0145	*0.091*	*0.089*
HOMA-B	123.31 (75.64,181.55)	104.67 (72.25,156.83)	95.79 (74.31,147.13)	−0.0344	0.0157	**0.028**	**0.025**
Gutt-ISI	635.63(490.54,793.14)	584.37(487.26,741.31)	602.96(473.75,750.12)	−0.0079	0.0079	0.317	0.238

Data are shown as mean±SD or median (interquartile range).

BMI, body mass index; HDL-C, high-density lipoprotein cholesterol; LDL-C, low-density lipoprotein cholesterol; HOMA-IR, homeostasis assessment model of insulin resistance;

HOMA-B, homeostasis assessment model of β-cell function; Gutt-ISI, insulin sensitivity index proposed by Gutt.

*p* values <0.05 are shown in bold, *p* values <0.1 are shown in italics.

*Adjusted for age, gender and BMI.

## Discussion

In the present study, three tagging SNPs in the region of the *FNDC5* gene, which encodes the myokine irisin, were tested for their association with type 2 diabetes and related quantitative traits in a Chinese Han population. We found that the effect of the common SNP rs16835198 on fasting insulin was significantly modified by BMI.

Irisin was originally characterized as a regulator of brown-fat-like development in specific depots of white adipose tissue, whose overexpression in mice resulted in increased energy expenditure, improved glucose tolerance, and modest but significant weight loss [8]. Irisin has since been linked to a variety of disorders, including obesity, anorexia nervosa, insulin sensitivity, nonalcoholic fatty liver disease and chronic kidney disease [27]–[30]. Despite these reports, to the best of our knowledge, the relationship between variants of *FNDC5* and the risk of type 2 diabetes and related clinical parameters in the Chinese population has not been previously evaluated. Here, we found that rs16835198 was significantly associated with the levels of fasting insulin in normal weight subjects without diabetes, and that the presence of the major allele G had an insulin-desensitizing effect on these individuals. Nevertheless, an opposite trend towards association of rs16835198 with fasting insulin and HOMA-IR levels was observed in overweight/obese controls. That is to say, the association of fasting insulin levels with this SNP in specific BMI-defined subgroups indicates that BMI contributes to the effect of this SNP on glycolipid metabolism. Moreover, we also detected that with each additional copy of the major allele G of rs16835198, HOMA-β cell function levels decreased significantly in overweight/obese controls, which means this SNP also influence β cell function in this part of subjects. However, so far, there is no unambiguous functional study focus on whether BMI modified the association between variants within *FNDC5* and glycolipid metabolism. The exact mechanisms underlying the results should be worthy for further investigation.

In contrast, a previous study in Southern German Caucasians indicated that the effect of SNPs rs16835198 and rs726344 on insulin sensitivity and resistance was not BMI-dependent [14]. However, rs726344 was excluded from our study, due to its extremely low minor allele frequency (MAF = 0), as estimated from the HapMap Chinese database. Additionally, we also observed that rs3480 had a trend towards association with HDL and LDL levels in the overweight/obesity participants (Table S3 in File S1), rather than fasting insulin values, which is not consistent with the latest study performed in Japan [15]. These results reiterate the relationship of genetic architecture with ethnicity, and further highlight the importance of perform genetic analyses in multiple populations with distinct genetic backgrounds.

The present study has some limitations. Firstly, we didn’t detect the circulating irisin levels in our samples, which may enhance the conclusion and make the article more interesting. Secondly, as our patients with diabetes were not newly diagnosed and were treated with anti-diabetic drugs and/or insulin, we cannot perform the association between the SNPs in *FNDC5* and clinical parameters in patients. Thirdly, due to lacking of functional study, the inner mechanism is still unclear. Therefore, it is imperative to extend studies of the influence of *FNDC5* variants on type 2 diabetes and metabolic traits in other Chinese populations.

## Conclusion

Our data suggest that the contribution of the common *FNDC5* SNP rs16835198 to fasting insulin is significantly modified by BMI. Further investigations with larger East Asian origin populations are necessary to confirm and further characterize our observations.

## Supporting Information

File S1 **Table S1,** association between SNPs in *FNDC5* and clinical characteristics in the normal glucose tolerant group. **Table S2,** Association analyses of the rs3480 and rs1570569 genotypes with clinical characteristics in normal weight controls. **Table S3,** Association analyses of the rs3480 and rs1570569 genotypes with clinical characteristics in overweight and obese individuals.
(DOCX)
