# Supplementary material for: An Interaction between a FNDC5 Variant and Obesity Modulates Glucose Metabolism in a Chinese Han Population
Source: PLoS One. 2014 Nov 4;9(11):e109957. doi: 10.1371/journal.pone.0109957 (PMC4219676; doi:10.1371/journal.pone.0109957)
Supplement: File S1 — Table S1, association between SNPs in FNDC5 and clinical characteristics in the normal glucose tolerant group. Table S2, Association analyses of the rs3480 and rs1570569 genotypes with clinical characteristics in normal weight controls. Table S3, Association analyses of the rs3480 and rs1570569 genotypes with clinical characteristics in overweight and obese individuals. (DOCX) [file pone.0109957.s001.docx]

**TableS1.** Association between SNPs in *FNDC5* and clinical characteristics in the normal glucose tolerant group

| **rs16835198** | **TT(n=768)** | **GT (n=1735)** | **GG (n=899)** | **β** | **SE** | ***p*** | ***p**** |
| --- | --- | --- | --- | --- | --- | --- | --- |
| Age (years) | 51.41±14.71 | 51.49±14.00 | 51.19±14.88 | -0.1172 | 0.3531 | 0.740 | / |
| BMI (kg/m^2^) | 23.15 (21.22,25.39) | 23.31 (21.34,25.59) | 23.22(21.22,25.39) | -0.0388 | 0.0792 | 0.625 | / |
| Fasting plasma glucose(mmol/L) | 5.04 (4.70,5.40) | 5.01 (4.69,5.40) | 5.04 (4.70,5.40) | -0.00004 | 0.0011 | 0.964 | 0.923 |
| 2-h plasma glucose (mmol/L) | 5.52 (4.60,6.30) | 5.43 (4.60,6.32) | 5.35 (4.60,6.27) | -0.0015 | 0.0023 | 0.524 | 0.581 |
| Fasting insulin (mU/L) | 6.22(4.29,9.08) | 6.20 (4.35,8.53) | 6.08 (4.41,8.61) | 0.0030 | 0.0078 | 0.699 | 0.595 |
| 2-h insulin (mU/L) | 26.77 (16.52,47.10) | 28.78 (14.93,46.70) | 26.61 (15.13,45.91) | -0.0014 | 0.0107 | 0.894 | 0.943 |
| Total cholesterol (mmol/L) | 4.70 (4.05,5.40) | 4.70 (4.04,5.35) | 4.65 (4.01,5.30) | -0.0018 | 0.0024 | 0.448 | 0.508 |
| Triglyceride (mmol/L) | 1.25(0.88,1.82) | 1.25 (0.87,1.82) | 1.25 (0.85,1.82) | -0.0049 | 0.0058 | 0.403 | 0.495 |
| HDL-C (mmol/L) | 1.34(1.13,1.51) | 1.32 (1.13,1.51) | 1.33(1.12,1.53) | 0.0016 | 0.0024 | 0.518 | 0.579 |
| LDL-C (mmol/L) | 3.06 (2.48,3.60) | 3.06 (2.53,3.63) | 2.98 (2.41,3.60) | -0.0042 | 0.0033 | 0.200 | 0.228 |
| HOMA-IR | 1.32 (0.94,1.96) | 1.35 (0.92,1.86) | 1.31 (0.93,1.89) | 0.0031 | 0.0081 | 0.699 | 0.590 |
| HOMA-B | 89.67 (60.61,137.06) | 90.78 (62.70,137.14) | 89.31 (63.45,132.24) | -0.0002 | 0.0087 | 0.979 | 0.953 |
| Gutt-ISI | 669.71 (544.11,867.23) | 657.47 (537.69,856.17) | 673.75(548.54,855.98) | -0.0009 | 0.0043 | 0.840 | 0.889 |

Data are shown as mean±SD or median (interquartile range)

BMI, body mass index; HDL-C, high-density lipoproteincholesterol; LDL-C, low-density lipoproteincholesterol; HOMA-IR, homeostasis assessment model of insulin resistance;

HOMA-B, homeostasis assessment model of β-cell function; Gutt-ISI, insulin sensitivity index proposed by Gutt.

*Adjusted for age, gender and BMI.

| **rs3480** | **GG (n=225)** | **AG (n=1306)** | **AA(n=1874)** | **β** | **SE** | ***p*** | ***p**** |
| --- | --- | --- | --- | --- | --- | --- | --- |
| Age (years) | 50.37±14.97 | 51.57±14.26 | 51.43±14.41 | 0.2023 | 0.3990 | 0.612 | / |
| BMI (kg/m^2^) | 23.12 (21.25,25.48) | 23.25(21.23,25.56) | 23.23 (21.28,25.46) | 0.1080 | 0.0895 | 0.228 | / |
| Fasting plasma glucose(mmol/L) | 5.05 (4.73,5.50) | 5.02 (4.70,5.37) | 5.02 (4.70,5.40) | -0.0011 | 0.0012 | 0.399 | 0.313 |
| 2-h plasma glucose (mmol/L) | 5.31 (4.50,6.20) | 5.40 (4.60,6.30) | 5.50 (4.60,6.30) | 0.0031 | 0.0026 | 0.236 | 0.346 |
| Fasting insulin (mU/L) | 6.23(4.39,8.84) | 6.08 (4.41,8.60) | 6.22 (4.33,8.65) | -0.0024 | 0.0087 | 0.780 | 0.622 |
| 2-h insulin (mU/L) | 29.73(16.15,49.74) | 27.26 (15.98,45.53) | 28.21(15.03,47.57) | -0.0066 | 0.0120 | 0.580 | 0.458 |
| Total cholesterol (mmol/L) | 4.70 (4.12,5.30) | 4.70 (4.05,5.35) | 4.70 (4.03,5.35) | -0.0010 | 0.0027 | 0.707 | 0.504 |
| Triglyceride (mmol/L) | 1.28 (0.91,1.86) | 1.25 (0.86,1.79) | 1.260.87,1.84) | 0.0037 | 0.0066 | 0.576 | 0.823 |
| HDL-C (mmol/L) | 1.33 (1.12,1.56) | 1.31(1.13,1.51) | 1.34(1.13,1.51) | 0.0004 | 0.0027 | 0.884 | 0.737 |
| LDL-C (mmol/L) | 3.01 (2.45,3.71) | 3.05 (2.50,3.63) | 3.02 (2.49,3.60) | -0.0014 | 0.0037 | 0.705 | 0.518 |
| HOMA-IR | 1.34(0.92,1.93) | 1.32 (0.92,1.89) | 1.33 (0.93,1.91) | -0.0040 | 0.0091 | 0.658 | 0.499 |
| HOMA-B | 88.19(61.68,127.18) | 90.65 (63.20,136.25) | 89.87 (62.60, 136.84) | 0.0036 | 0.0098 | 0.712 | 0.795 |
| Gutt-ISI | 669.72(527.83,863.67) | 667.64(550.69,849.47) | 662.75(537.49,872.43) | 0.0023 | 0.0051 | 0.655 | 0.526 |

Data are shown as mean±SD or median (interquartile range)

BMI, body mass index; HDL-C, high-density lipoproteincholesterol; LDL-C, low-density lipoproteincholesterol; HOMA-IR, homeostasis assessment model of insulin resistance;

HOMA-B, homeostasis assessment model of β-cell function; Gutt-ISI, insulin sensitivity index proposed by Gutt.

*Adjusted for age, gender and BMI.

| **rs1570569** | **TT (n=153)** | **GT (n=1122)** | **GG(n=2121)** | **β** | **SE** | ***p*** | ***p**** |
| --- | --- | --- | --- | --- | --- | --- | --- |
| Age (years) | 50.92±14.67 | 51.39±14.03 | 51.44±15.58 | 0.1362 | 0.4277 | 0.750 | / |
| BMI (kg/m^2^) | 23.05 (21.19,25.51) | 23.27 (21.29,25.54) | 23.23 (21.28,25.46) | 0.0906 | 0.0957 | 0.344 | / |
| Fasting plasma glucose(mmol/L) | 5.10 (4.70,5.50) | 5.00 (4.67,5.36) | 5.03 (4.70,5.40) | -0.00007 | 0.0013 | 0.958 | 0.863 |
| 2-h plasma glucose (mmol/L) | 5.40 (4.52,6.27) | 5.40 (4.60,6.29) | 5.47 (4.60,6.33) | 0.0030 | 0.0028 | 0.290 | 0.390 |
| Fasting insulin (mU/L) | 6.34 (4.70,8.95) | 5.99 (4.34,8.56) | 6.22 (4.33,8.66) | -0.0028 | 0.0094 | 0.764 | 0.944 |
| 2-h insulin (mU/L) | 29.09 (16.60,53.67) | 27.62 (16.05,45.46) | 27.99 (15.06,47.14) | -0.0066 | 0.0129 | 0.609 | 0.477 |
| Total cholesterol (mmol/L) | 4.74 (4.15,5.35) | 4.67 (4.01,5.34) | 4.70 (4.04,5.35) | -0.0003 | 0.0029 | 0.923 | 0.750 |
| Triglyceride (mmol/L) | 1.35 (0.94,1.91) | 1.24 (0.87,1.76) | 1.25 (0.86,1.85) | -0.0004 | 0.0071 | 0.959 | 0.739 |
| HDL-C (mmol/L) | 1.30 (1.11,1.56) | 1.31 (1.11,1.50) | 1.34(1.14,1.52) | 0.0041 | 0.0029 | 0.158 | 0.106 |
| LDL-C (mmol/L) | 2.97 (2.45,3.73) | 3.05 (2.48,3.62) | 3.03 (2.50,3.60) | 0.0004 | 0.0039 | 0.925 | 0.928 |
| HOMA-IR | 1.30 (1.11,1.56) | 1.31 (1.11,1.50) | 1.34 (1.14,1.52) | -0.0029 | 0.0098 | 0.768 | 0.594 |
| HOMA-B | 91.28 (66.00,127.18) | 90.33 (62.81,136.25) | 89.85 (61.90,136.07) | -0.0037 | 0.010 | 0.726 | 0.637 |
| Gutt-ISI | 664.51(520.13,837.86) | 666.95(552.78,849.47) | 665.16(538.66,870.32) | 0.0010 | 0.0056 | 0.863 | 0.692 |

Data are shown as mean±SD or median (interquartile range)

BMI, body mass index; HDL-C, high-density lipoproteincholesterol; LDL-C, low-density lipoproteincholesterol; HOMA-IR, homeostasis assessment model of insulin resistance;

HOMA-B, homeostasis assessment model of β-cell function; Gutt-ISI, insulin sensitivity index proposed by Gutt.

*Adjusted for age, gender and BMI.

**Table S2**. Association analyses of the rs3480 and rs1570569 genotypes with clinical characteristics in normal weight controls

| **rs3480** | **GG (n=160)** | **AG (n=913)** | **AA(n=1308)** | **β** | **SE** | ***p*** | ***p**** |
| --- | --- | --- | --- | --- | --- | --- | --- |
| Age (years) | 49.36±15.51 | 51.11±14.93 | 51.28±14.73 | -0.1631 | 0.4915 | 0.740 | / |
| BMI (kg/m^2^) | 21.94 (20.56,23.29) | 22.14 (20.52,23.44) | 22.07 (20.57,23.48) | 0.0625 | 0.0662 | 0.346 | / |
| Fasting plasma glucose(mmol/L) | 5.00 (4.61,5.40) | 5.00 (4.64,5.33) | 5.00 (4.65,5.38) | -0.0005 | 0.0015 | 0.729 | 0.693 |
| 2-h plasma glucose (mmol/L) | 5.31 (4.50,6.10) | 5.30 (4.60,6.14) | 5.31 (4.51,6.20) | 0.0014 | 0.0031 | 0.660 | 0.648 |
| Fasting insulin (mU/L) | 5.87 (4.21,7.75) | 5.59 (3.94,7.79) | 5.63 (4.03,7.73) | -0.0058 | 0.0101 | 0.567 | 0.573 |
| 2-h insulin (mU/L) | 28.77 (15.71,44.28) | 24.95 (14.14,39.34) | 24.77 (13.49,42.61) | -0.0113 | 0.0139 | 0.417 | 0.475 |
| Total cholesterol (mmol/L) | 4.64 (3.95,5.30) | 4.62 (4.00,5.30) | 4.60 (3.96,5.29) | 0.0006 | 0.0033 | 0.844 | 0.745 |
| Triglyceride (mmol/L) | 1.16 (0.81,1.68) | 1.11 (0.79,1.60) | 1.14 (0.82,1.67) | 0.0054 | 0.0075 | 0.469 | 0.574 |
| HDL-C (mmol/L) | 1.36 (1.16,1.59) | 1.36 (1.18,1.56) | 1.36(1.20,1.55) | 0.0033 | 0.0032 | 0.290 | 0.146 |
| LDL-C (mmol/L) | 2.92 (2.37,3.59) | 3.00 (2.41,3.57) | 2.98 (2.44,3.51) | 0.0024 | 0.0044 | 0.584 | 0.531 |
| HOMA-IR | 1.25 (0.91,1.73) | 1.22 (0.85,1.65) | 1.20 (0.85,1.71) | -0.0064 | 0.0105 | 0.542 | 0.544 |
| HOMA-B | 88.15 (61.56,127.18) | 84.58 (59.76,124.62) | 83.51 (59.05,127.41) | -0.0035 | 0.0116 | 0.763 | 0.772 |
| Gutt-ISI | 694.72(563.23,927.26) | 706.96(582.08,876.22) | 697.42(569.54,904.90) | 0.0037 | 0.0006 | 0.550 | 0.635 |

Data are shown as mean±SD or median (interquartile range)

BMI, body mass index; HDL-C, high-density lipoproteincholesterol; LDL-C, low-density lipoproteincholesterol; HOMA-IR, homeostasis assessment model of insulin resistance;

HOMA-B, homeostasis assessment model of β-cell function; Gutt-ISI, insulin sensitivity index proposed by Gutt.

*p* values <0.05 are shown in bold.

*Adjusted for age, gender and BMI.

| **rs1570569** | **TT (n=110)** | **GT (n=781)** | **GG(n=1486)** | **β** | **SE** | ***p*** | ***p**** |
| --- | --- | --- | --- | --- | --- | --- | --- |
| Age (years) | 50.47±14.81 | 50.90 ±14.75 | 50.31±14.95 | -0.3628 | 0.5260 | 0.490 | / |
| BMI (kg/m^2^) | 21.86 (20.45,23.23) | 22.15 (20.55,23.42) | 22.04 (20.56,23.49) | 0.0646 | 0.0708 | 0.362 | / |
| Fasting plasma glucose(mmol/L) | 5.02 (4.60,5.50) | 5.00 (4.60,5.32) | 5.00 (4.65,5.39) | -0.0001 | 0.0016 | 0.953 | 0.928 |
| 2-h plasma glucose (mmol/L) | 5.36 (4.53,6.13) | 5.31 (4.60,6.16) | 5.30 (4.51,6.20) | -0.00001 | 0.0033 | 0.997 | 0.980 |
| Fasting insulin (mU/L) | 5.96 (4.39,7.61) | 5.60 (3.99,7.74) | 5.61 (4.02,7.79) | -0.0083 | 0.0108 | 0.441 | 0.457 |
| 2-h insulin (mU/L) | 28.12 (15.71,52.90) | 24.97 (14.01,41.37) | 24.09 (13.78,41.65) | -0.0139 | 0.0150 | 0.352 | 0.434 |
| Total cholesterol (mmol/L) | 4.65 (3.83,5.40) | 4.60 (3.96,5.29) | 4.61 (4.00,5.29) | 0.0016 | 0.0035 | 0.655 | 0.502 |
| Triglyceride (mmol/L) | 1.28 (0.87,1.74) | 1.10 (0.79,1.56) | 1.13 (0.81,1.67) | 0.0010 | 0.0080 | 0.901 | 0.973 |
| HDL-C (mmol/L) | 1.32 (1.16,1.59) | 1.35 (1.15,1.55) | 1.36(1.20,1.55) | 0.0059 | 0.0034 | *0.078* | **0.033** |
| LDL-C (mmol/L) | 2.91 (2.36,3.70) | 3.00 (2.40,3.56) | 2.98 (2.44,3.51) | 0.0045 | 0.0047 | 0.338 | 0.247 |
| HOMA-IR | 1.27 (0.92,1.69) | 1.21 (0.86,1.63) | 1.21 (0.84,1.71) | -0.0082 | 0.0112 | 0.467 | 0.483 |
| HOMA-B | 87.58 (62.45,127.18) | 86.09 (60.12,125.76) | 83.22 (58.96,124.57) | -0.0109 | 0.0124 | 0.381 | 0.388 |
| Gutt-ISI | 683.25(538.64,927.26) | 698.96(577.72,885.78) | 698.62(573.53,903.24) | 0.0039 | 0.0066 | 0.863 | 0.651 |

Data are shown as mean±SD or median (interquartile range)

BMI, body mass index; HDL-C, high-density lipoproteincholesterol; LDL-C, low-density lipoproteincholesterol; HOMA-IR, homeostasis assessment model of insulin resistance;

HOMA-B, homeostasis assessment model of β-cell function; Gutt-ISI, insulin sensitivity index proposed by Gutt.

*p* values <0.05 are shown in bold, *p* values <0.1 are shown in italics.

*Adjusted for age, gender and BMI.

**Table S3.** Association analyses of the rs3480 and rs1570569 genotypes with clinical characteristics in overweight and obese individuals

| **rs3480** | **GG (n=65)** | **AG (n=393)** | **AA(n=563)** | **β** | **SE** | ***p*** | ***p**** |
| --- | --- | --- | --- | --- | --- | --- | --- |
| Age (years) | 52.88±13.33 | 52.63±12.51 | 54.06±13.21 | 1.0100 | 0.6599 | 0.126 | / |
| BMI (kg/m^2^) | 26.30 (25.64,27.81) | 26.79 (25.81,28.13) | 26.84 (25.82,28.26) | 0.1591 | 0.1060 | 0.134 | / |
| Fasting plasma glucose(mmol/L) | 5.20 (4.90,5.60) | 5.10 (4.77,5.40) | 5.10 (4.70,5.42) | -0.0022 | 0.0022 | 0.304 | 0.226 |
| 2-h plasma glucose (mmol/L) | 5.40 (4.60,6.60) | 5.67 (4.75,6.54) | 5.80 (4.90,6.64) | 0.0069 | 0.0047 | 0.140 | 0.282 |
| Fasting insulin (mU/L) | 7.39 (5.66,10.18) | 7.79 (5.21,11.07) | 7.44 (5.39,10.38) | 0.0049 | 0.0101 | 0.713 | 0.901 |
| 2-h insulin (mU/L) | 34.05 (24.90,60.18) | 33.72 (19.74,57.52) | 36.66 (19.19,59.07) | 0.0070 | 0.0220 | 0.794 | 0.969 |
| Total cholesterol (mmol/L) | 4.90 (4.49,5.28) | 4.86 (4.20,5.55) | 4.85 (4.18,5.50) | -0.0051 | 0.0047 | 0.273 | 0.134 |
| Triglyceride (mmol/L) | 1.62 (1.07,2.29) | 1.60 (1.12,2.24) | 1.61 (1.11,2.26) | -0.0016 | 0.0121 | 0.893 | 0.990 |
| HDL-C (mmol/L) | 1.26 (1.09,1.50) | 1.25 (1.08,1.41) | 1.25(1.01,1.44) | -0.0060 | 0.0051 | 0.232 | *0.081* |
| LDL-C (mmol/L) | 3.32 (2.73,3.78) | 3.19 (2.64,3.80) | 3.18(2.64,3.80) | -0.0106 | 0.0066 | 0.109 | *0.057* |
| HOMA-IR | 1.72(1.19,2.31) | 1.74 (1.09,2.54) | 1.66 (1.15,2.37) | 0.0021 | 0.0162 | 0.897 | 0.894 |
| HOMA-B | 89.73 (73.02,126.29) | 109.63 (75.46,162.88) | 106.40 (72.09,163.50) | 0.0201 | 0.0175 | 0.252 | 0.312 |
| Gutt-ISI | 606.70(463.70,750.12) | 610.49(494.93,750.20) | 582.61(482.43,758.82) | -0.0017 | 0.0088 | 0.849 | 0.856 |

Data are shown as mean±SD or median (interquartile range)

BMI, body mass index; HDL-C, high-density lipoproteincholesterol; LDL-C, low-density lipoproteincholesterol; HOMA-IR, homeostasis assessment model of insulin resistance;

HOMA-B, homeostasis assessment model of β-cell function; Gutt-ISI, insulin sensitivity index proposed by Gutt.

*p* values <0.1are shown in italics.

*Adjusted for age, gender and BMI.

| **rs1570569** | **TT (n=43)** | **GT (n=341)** | **GG(n=632)** | **β** | **SE** | ***p*** | ***p**** |
| --- | --- | --- | --- | --- | --- | --- | --- |
| Age (years) | 52.05±14.41 | 52.53±12.16 | 54.04±13.26 | 1.2984 | 0.7090 | *0.067* | / |
| BMI (kg/m^2^) | 26.51 (25.70,27.99) | 26.65 (25.73,28.13) | 26.84 (25.85,28.25) | 0.1431 | 0.1133 | 0.207 | / |
| Fasting plasma glucose(mmol/L) | 5.20 (4.80,5.47) | 5.03 (4.74,5.42) | 5.10 (4.70,5.44) | 0.0001 | 0.0023 | 0.960 | 0.886 |
| 2-h plasma glucose (mmol/L) | 5.44 (4.50,6.83) | 5.60 (4.69,6.50) | 5.80 (4.90,6.68) | 0.0101 | 0.0050 | **0.047** | 0.112 |
| Fasting insulin (mU/L) | 7.46 (5.80,10.33) | 7.77 (4.94,10.75) | 7.48 (5.41,10.40) | 0.0091 | 0.0171 | 0.593 | 0.775 |
| 2-h insulin (mU/L) | 34.05 (19.62,10.33) | 33.07 (19.47,57.79) | 32.27 (19.40,59.07) | 0.0107 | 0.0239 | 0.655 | 0.938 |
| Total cholesterol (mmol/L) | 4.86 (4.50,5.27) | 4.84 (4.20,5.55) | 4.88 (4.20,5.50) | -0.0047 | 0.0050 | 0.344 | 0.167 |
| Triglyceride (mmol/L) | 1.62 (1.01,2.45) | 1.60 (1.13,2.27) | 1.62 (1.11,2.25) | -0.0041 | 0.0130 | 0.754 | 0.813 |
| HDL-C (mmol/L) | 1.20 (1.07,1.43) | 1.24 (1.07,1.41) | 1.27(1.02,1.44) | 0.0001 | 0.0054 | 0.985 | 0.660 |
| LDL-C (mmol/L) | 3.31 (2.72,3.79) | 3.21 (2.67,3.79) | 3.17 (2.63,3.80) | -0.0095 | 0.0071 | 0.177 | *0.095* |
| HOMA-IR | 1.77 (1.28,2.48) | 1.68 (1.04,2.49) | 1.68 (1.16,2.38) | 0.0087 | 0.0177 | 0.621 | 0.823 |
| HOMA-B | 102.38 (77.79,132.92) | 108.00(74.95,156.93) | 106.40 (72.25,163.50) | 0.0123 | 0.0191 | 0.519 | 0.600 |
| Gutt-ISI | 622.84(440.59,763.65) | 619.67(496.33,763.11) | 581.22(580.89,746.42) | -0.0059 | 0.0095 | 0.539 | 0.865 |

Data are shown as mean±SD or median (interquartile range)

BMI, body mass index; HDL-C, high-density lipoproteincholesterol; LDL-C, low-density lipoproteincholesterol; HOMA-IR, homeostasis assessment model of insulin resistance;

HOMA-B, homeostasis assessment model of β-cell function; Gutt-ISI, insulin sensitivity index proposed by Gutt.

*p* values <0.05 are shown in bold, *p* values <0.1 are shown in italics.

*Adjusted for age, gender and BMI.
